# Isolation and characterization of a minimal building block of polyubiquitin fibrils

**DOI:** 10.1038/s41598-018-21144-z

**Published:** 2018-02-09

**Authors:** Daichi Morimoto, Erik Walinda, Mayo Shinke, Kenji Sugase, Masahiro Shirakawa

**Affiliations:** 1 Department of Molecular Engineering, Graduate School of Engineering, Kyoto University, Kyoto-Daigaku Katsura, Nishikyo-Ku, Kyoto, 615-8510 Japan; 2 Department of Molecular and Cellular Physiology, Graduate School of Medicine, Kyoto University, Yoshida Konoe-cho, Sakyo-Ku, Kyoto, 606-8501 Japan

## Abstract

As a posttranslational modifier, polyubiquitin is involved in the regulation of diverse intracellular processes; however, it is also found in pathological protein aggregates associated with Alzheimer’s disease and other neurodegenerative disorders. We previously observed that various types of polyubiquitin can form amyloid-like fibrils; however, the structural properties of these polyubiquitin fibrils have not been examined at an atomic level. Here we demonstrate that a soluble intermediate species can be extracted from disulfide-conjugated diubiquitin fibrils after cleaving the disulfide bonds in the fibrils. This newly discovered molecule is structurally and physicochemically distinguishable from native ubiquitin. In addition, it is thermodynamically metastable, as demonstrated by real-time NMR measurements. Collectively, our results suggest that the fibril-derived molecule is a minimal building block of polyubiquitin fibrils that reflects their structural and physicochemical properties.

## Introduction

Amyloid formation is associated with various intractable human diseases such as neurodegenerative diseases, cancer, and type II diabetes^1^. Many kinds of peptides and proteins have been found to form amyloid fibrils, one of which is polyubiquitin^2^. Although ubiquitin is a relatively rigid intracellular protein that functions as a posttranslational modifier, it is often found in inclusion bodies associated with neurodegenerative disorders such as Alzheimer’s disease and Parkinson’s disease^3,4^. We previously showed that the thermodynamic stability of ubiquitin decreases with polymerization, and that polyubiquitin forms amyloid-like fibrils when heat or shear stress is applied^2^. In addition, we recently found that the covalent conjugation of ubiquitin molecules (ubiquitylation) to proteins induces structural fluctuations, resulting in a decreased in thermodynamic stability^5^. Thus, the formation of an (iso-)peptide bond between ubiquitin units in polyubiquitin seems to markedly alter the physicochemical properties of the ubiquitin molecules, leading to the production of amyloid-like fibrils. However, the mechanism underlying the ubiquitylation-driven formation of fibrils and the detailed structure of these fibrils remain unclear.

Diubiquitin, the shortest possible form of polyubiquitin, offers a simple molecule with which to study the mechanism of fibril formation. Diubiquitin is formed by covalent conjugation of the carboxyl group of the C-terminus of one ubiquitin molecule to the amino group of either the N-terminus or an internal lysine residues of a second ubiquitin molecule. The tertiary structures of the two ubiquitin moieties in diubiquitin are almost indistinguishable from that of monoubiquitin^6^; however, diubiquitin forms fibrillar aggregates under heat or shear stress, whereas monoubiquitin does not^2^. Therefore, we hypothesized that cleavage of the peptide bond connecting the two ubiquitin moieties in a fibril of diubiquitin might revert fibril formation to a state where soluble fibril-derived molecules can be isolated and studied by solution NMR methods.

To test this hypothesis, here we focused on fibrils of disulfide-conjugated diubiquitin. It is difficult to selectively cleave a single peptide bond of interest, but a disulfide bridge can be selectively cleaved by the addition of reducing agents under normal biochemical buffer conditions. We found that a soluble intermediate species can be extracted from disulfide-conjugated diubiquitin fibrils by the addition of dithiothreitol (DTT). The novel fibril-derived molecule reflects the structural and physicochemical properties of polyubiquitin fibrils rather than those of monoubiquitin, and can be regarded as a minimal building block of the fibrils.

## Results

### Preparation of soluble fibril-derived molecules

**Figure 1:**
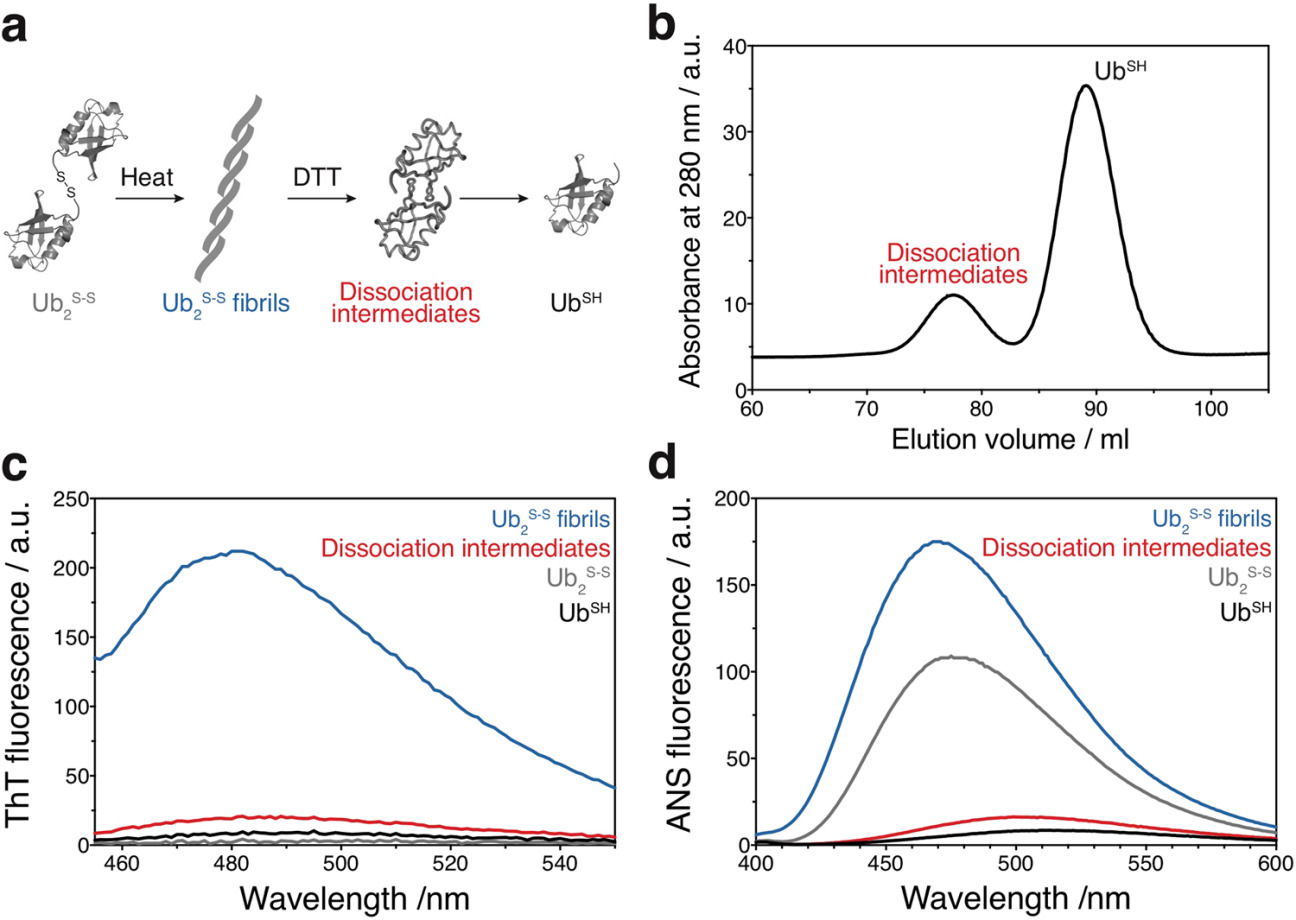
Isolation of a diubiquitin fibril-derived soluble intermediate. (**a**) Strategy for the preparation of soluble fibril-derived molecules by using disulfide-conjugated diubiquitin (Ub_2_^S-S^). Ub_2_^S-S^ was prepared by conjugation of the ubiquitin G76C mutant (Ub^SH^) via a disulfide bond. Ub_2_^S-S^ formed fibrillar aggregates after heat treatment. Subsequent cleavage of the disulfide bond by reducing agents resulted in dissociation of the fibrils, producing fibril-derived molecules (dissociation intermediates) in addition to Ub^SH^. (**b**) Size-exclusion elution profile of the solution obtained by treatment of the fibrils with reducing agent. The buffer contained 5 mM dithiothreitol (DTT). (**c**) Thioflavin T binding assay for Ub_2_^S-S^ fibril (blue), dissociation intermediates (red), Ub_2_^S-S^ (gray), and Ub^SH^ (black). (**d**) ANS binding assay for Ub_2_^S-S^ fibril (blue), dissociation intermediates (red), Ub_2_^S-S^ (gray), and Ub^SH^ (black).

Formation of a disulfide bridge between the C-terminal thiol group of a ubiquitin G76C mutant (Ub^SH^) and a newly introduced thiol group on a target protein is often utilized to mimic ubiquitylation^7^. Using this approach, we prepared disulfide-conjugated diubiquitin (Ub_2_^S-S^) using Ub^SH^ and production of Ub_2_^S-S^ was confirmed by mass spectrometry (Supporting Information (SI), Fig. S1). We observed no significant differences in ^1^H-^15^N HSQC spectra between Ub^SH^ and Ub_2_^S-S^ (SI, Figs S2 and S3). This suggests that no specific inter-subunit interactions were formed in Ub_2_^S-S^, as previously observed for K63-linked diubiquitin due to its extended conformation^8^. Because the disulfide bridge in this type of diubiquitin is formed between the C-termini of the two ubiquitin moieties, its overall physicochemical properties might differ from those of naturally occurring types of diubiquitin. Similar to three conventional types of diubiquitin (M1-, K48-, and K63-linked diubiquitin), however, fibrillar aggregates were formed upon heat treatment of Ub_2_^S-S^ (SI, Fig. S4; see also fibrils of the three types of diubiquitin in Morimoto, *et al*. Nat Commun 2015). Moreover, the fibrils stained positively for Thioflavin T (ThT), a dye commonly used to detect amyloid fibrils (Fig. 1c). Thus, Ub_2_^S-S^ mimics naturally occurring diubiquitin in terms of ThT-positive fibril formation.

Next, we investigated whether cleavage of the disulfide bond in Ub_2_^S-S^ fibrils would lead to dissociation of the fibril into smaller soluble fragments. Because monoubiquitin does not form fibrillar aggregates^2^, we considered that Ub_2_^S-S^ fibrils would dissociate into Ub^SH^ upon chemical cleavage of the disulfide bond. As expected, addition of the reducing agent DTT dissolved the Ub_2_^S-S^ fibrils; however, dissociation did not occur rapidly and more than 3 days of incubation was required to obtain a clear solution.

Using size-exclusion chromatography, we examined whether the solution contained soluble fragments extracted from the fibrils. Intriguingly, in addition to Ub^SH^ (second peak), we obtained a fibril-derived species (first peak; hereafter referred to as: “dissociation intermediates”) from the DTT-treated solution of Ub_2_^S-S^ fibrils (Fig. 1b and SI, Fig. S5 lower middle). The elution volume of the dissociation intermediates was very similar to that of Ub_2_^S-S^, but Ub_2_^S-S^ was expected to be completely reduced to Ub^SH^ owing to the high concentrations of DTT used. Indeed, we showed that Ub_2_^S-S^ was completely reduced and formed Ub^SH^ in the presence of DTT (SI, Fig. S5 upper middle). Therefore, all the cysteine residues in the dissociation intermediates appeared to be reduced by DTT. In addition, when the dissociation intermediates were isolated (first peak), they eluted at the same elution volume in a second size-exclusion step in the presence of DTT (SI, Fig. S5 bottom). Thus, the dissociation intermediates seemed to be a soluble oligomeric species derived from the insoluble fibrils.

We considered that these molecules might be held together by the non-covalent interactions that hold the Ub_2_^S-S^ fibrils together. On the basis of their elution volume, the dissociation intermediates have a hydrodynamic volume similar to that of Ub_2_^S-S^ (SI, Fig. S5 bottom); namely, twice the hydrodynamic volume of Ub^SH^. In addition, as compared with Ub^SH^ and Ub_2_^S-S^, the dissociation intermediates stained slightly more strongly for ThT (Fig. 1c), suggesting that they have partial amyloid-like properties. Unexpectedly, 8-anilino-1-naphthalenesulfonic acid (ANS), a fluorescent probe to quantify hydrophobic sites on proteins^9^, bound to Ub_2_^S-S^ as strongly as it bound to Ub_2_^S-S^ fibrils (Fig. 1d); however, the affinity of ANS seemed to be higher for the dissociation intermediates than for Ub^SH^. This observation indicated that the dissociation intermediates have a more hydrophobic surface as compared with monomeric ubiquitin. Because structural changes occurred during fibril formation, these data suggest that structural and physicochemical features of the Ub_2_^S-S^ fibrillar aggregates are retained in the soluble intermediate.

### Structural characterization of the dissociation intermediates of polyubiquitin fibrils

**Figure 2:**
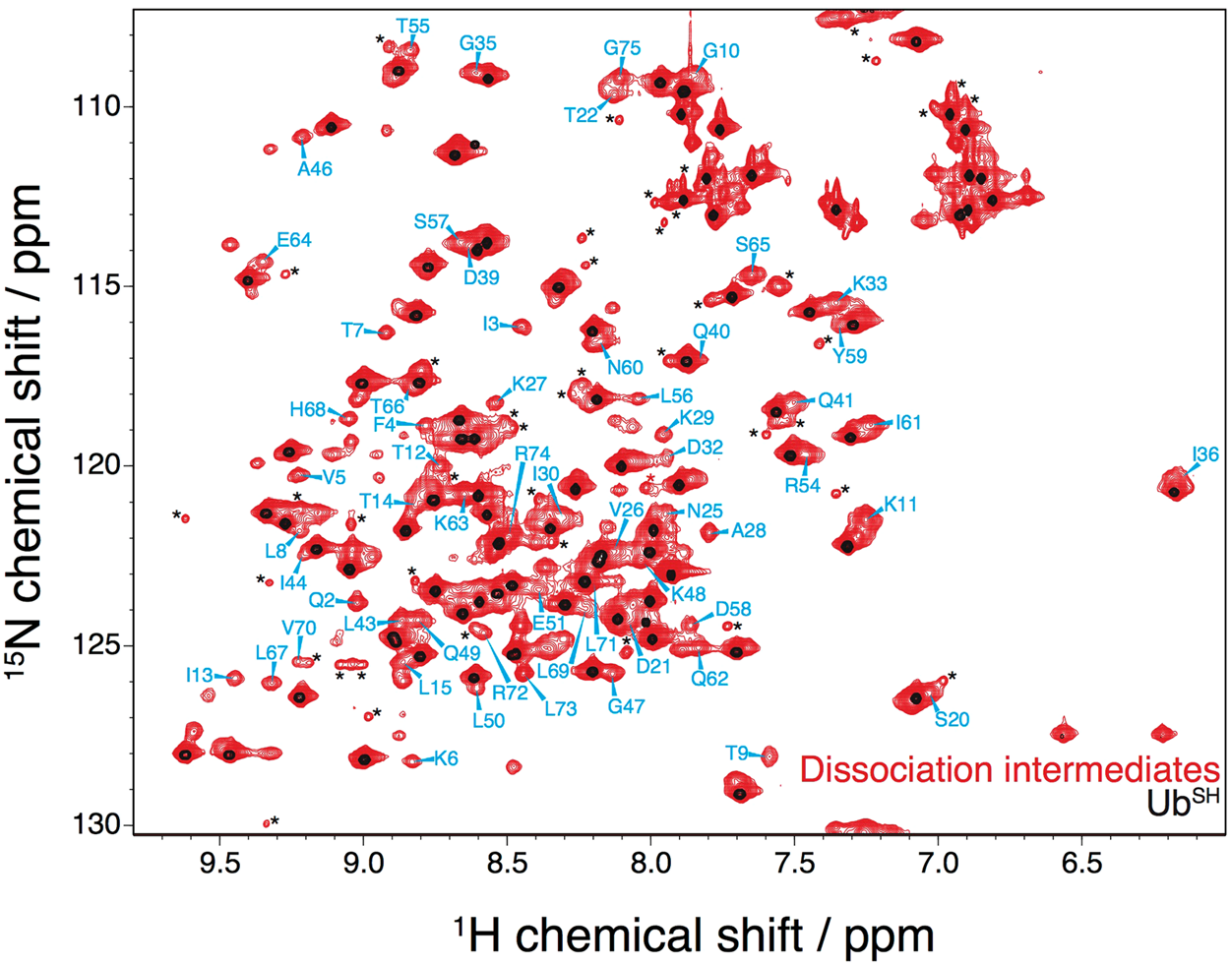
Characteristic NMR spectrum of dissociation intermediates. Shown are the ^1^H-^15^N HSQC spectra of the dissociation intermediates (red) and Ub^SH^ (black) at 285 K. Assigned peaks of the dissociation intermediates are indicated in cyan. Assigned chemical shifts are given in SI, Fig. S6. Asterisks indicate minor peaks that are also observed in the spectrum of Ub^SH^ (at 285 K). The cross-peaks of T9, S20, A46, and G47 are aliased. The contour levels in the spectrum of Ub^SH^ were chosen to provide a reference.

To investigate the structure of the fibril-derived molecule, we recorded its ^1^H-^15^N HSQC spectrum. Intriguingly, we detected a large number of cross-peaks in addition to the cross-peaks whose positions correspond to those of Ub^SH^ (Fig. 2). To the best of our knowledge, these minor peaks have not previously been reported for a soluble ubiquitin species. Because these were no differences in the chemical shifts of the major cross-peaks as compared with the spectrum of Ub^SH^, we considered that the sample of the isolated dissociation intermediates might contain Ub^SH^ owing to imperfect purification or partial conversion of dissociation intermediates to Ub^SH^. Extreme care was taken to collect only chromatography fractions that did not include Ub^SH^; nevertheless, we acquired the ^1^H-^15^N HSQC spectrum of numerous independent preparations of the dissociation intermediates, which all showed the same spectral pattern (Fig. 2). Owing to its low transverse relaxation rate, even a small amount of Ub^SH^ in the sample leads to strong cross-peaks in the spectrum. Indeed, line-broadening was observed in the minor cross-peaks relative to those of Ub^SH^. Therefore, based on the size exclusion chromatography analysis (Fig. 1b and SI, Fig. S5), we assumed that the dissociation intermediates represent a homodimer of a physicochemically different ubiquitin species.

**Figure 3:**
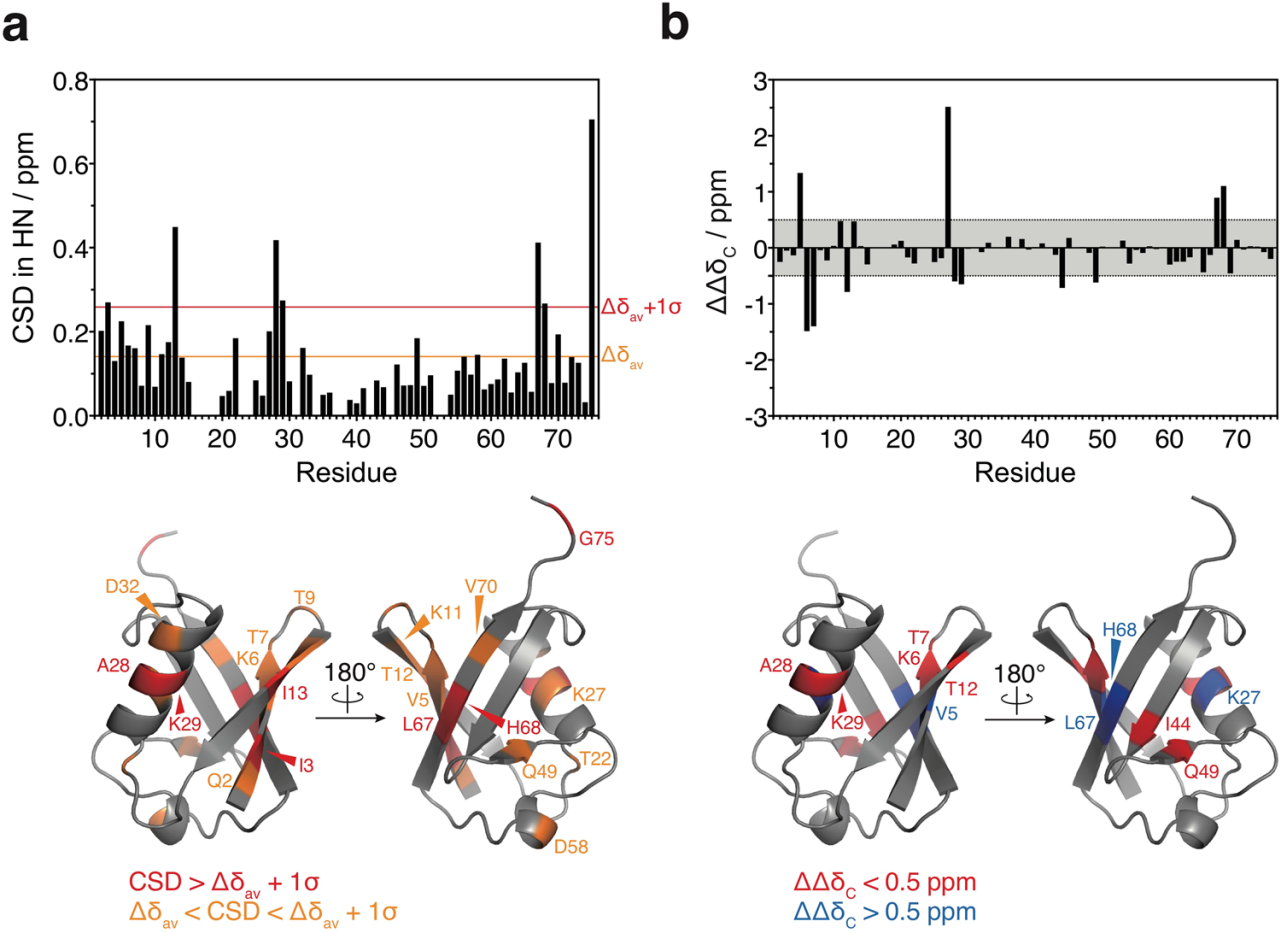
Structural comparison between dissociation intermediates and native mono-ubiquitin. (**a**) Top, ^1^H and ^15^N amide chemical shift differences (CSD) are obtained as weighted average chemical shift differences [Δδ_HN_^2^ + (0.2 Δδ_N_)^2^]^0.5^, where Δδ_HN_ and Δδ_N_ are ^1^H and ^15^N chemical shift differences between the dissociation intermediates and Ub^SH^. Orange and red lines indicate Δδ_av_ (average of CSD) and Δδ_av_ + 1σ (standard deviation), respectively. Bottom, residues showing Δδ_av_ < CSD < Δδ_av_ + 1σ (orange) and CSD > Δδ_av_ + 1σ (red) are mapped on the crystal structure of ubiquitin [Protein Data Bank (PDB) database entry 1UBQ]. (**b**) Top, differences in the degree of secondary structure formation are estimated from differences in the absolute values of the secondary chemical shifts between the dissociation intermediate and Ub^SH^: ΔΔδ_C_ = |Δδ_Cα_ − Δδ_Cβ_|_intermediate_ − |Δδ_Cα_ − Δδ_Cβ_|_UbSH_, where Δδ_Cα_ and Δδ_Cβ_ are the C_α_ and C_β_ chemical shift changes between observed and random-coil chemical shift values. Bottom, residues showing significant secondary chemical shift differences (>0.5 ppm or <−0.5 ppm) are mapped on the structure of ubiquitin (PDB database entry 1UBQ).

Although it was difficult to acquire C_α_ and C_β_ signals and to assign the resonances of several minor peaks due to severe line broadening and heavy peak overlap, HN, N, C_α_, and C_β_ chemical shifts of a large fraction of the dissociation intermediate signals were identified by sequential backbone assignment (Fig. 2 and SI, Fig. S6). Amide chemical shift differences between the dissociation intermediate and Ub^SH^ were observed mainly on three β-strands (β1: I3; β2: I13; β5: L67 and H68) and on the central part of the α-helix (A28 and K29) (Fig. 3a). These chemical shift differences might be caused by either inter-molecular interactions or changes in tertiary structure.

Because the dissociation intermediates have different structural features, as indicated by the ThT and ANS binding assays (Fig. 1c,d), we further analyzed the secondary chemical shifts of C_α_ and C_β_ to investigate conformational changes (SI, Fig. S7). Secondary chemical shifts are obtained as the difference between observed and random-coil chemical shifts of C_α_ and C_β_; thus, their absolute values reflect the degree of secondary structure formation. A decrease in secondary structure formation was detected on the edges of four β-strands (β1: K6 and T7; β2: T12; β3: I44; β4: Q49) and on the central part of the α-helix (A28 and K29) (Fig. 3b). This suggests that partial unfolding occurs in multiple regions of the structure of the dissociation intermediates. In contrast, an increase in secondary structure formation was observed on the central parts of secondary structure elements (β1: V5; α1: K27; β5: L67 and H68). Collectively, the observed amide chemical shift differences on the edges of β-strands and the center of the α-helix may be caused not only by inter-molecular interactions, but also by changes in secondary structure; by contrast, the changes in other regions of the three β-strands (β1, β2, and β5) may be mainly due to inter-molecular interactions. Therefore, the dissociation intermediates may consist of two partially unfolded ubiquitin molecules associating via inter-molecular interactions on the three β-strands.

### Thermodynamically unstable dissociation intermediates

**Figure 4:**
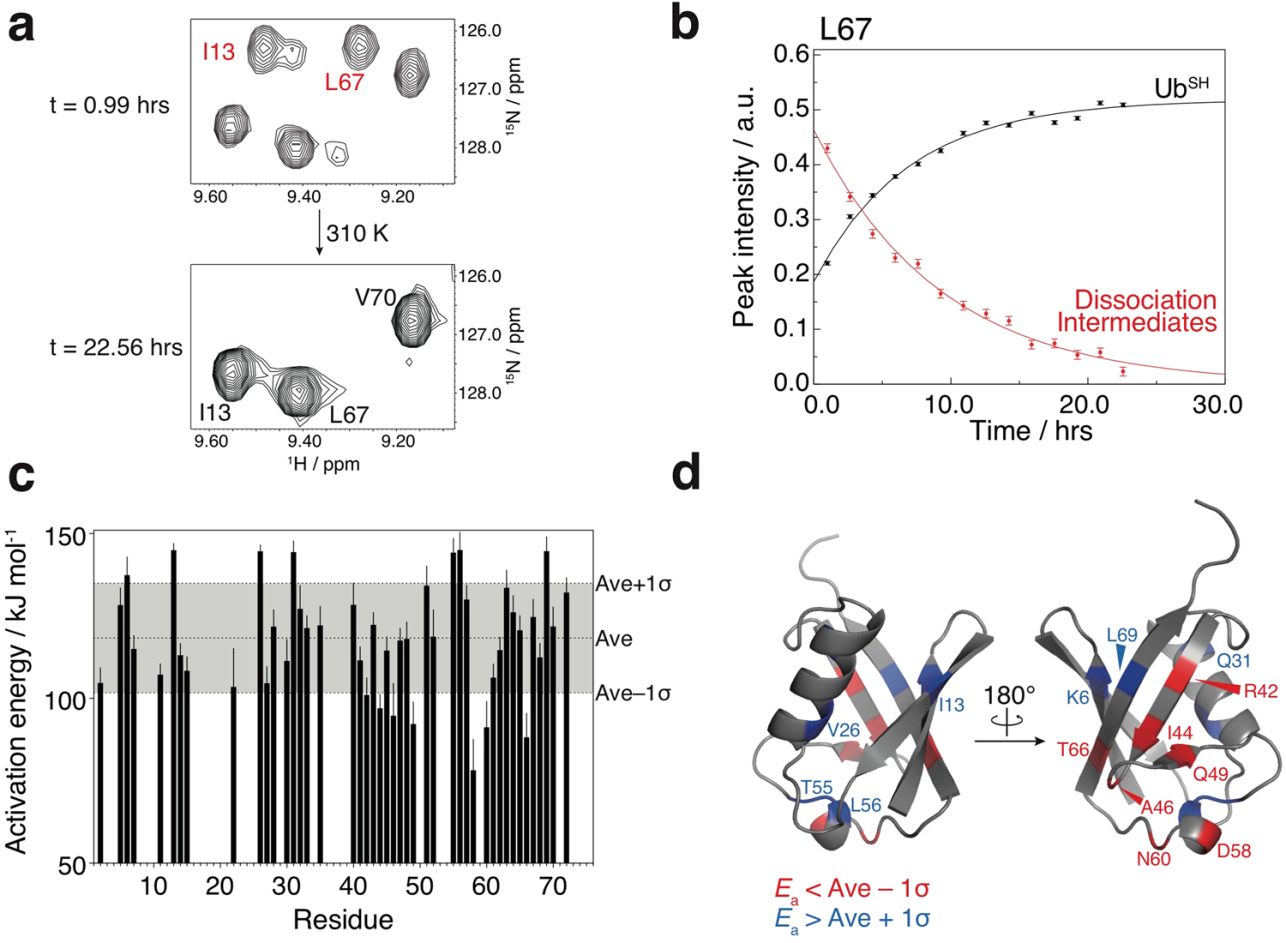
Thermodynamically metastable properties of dissociation intermediates. (**a**) Time-dependent ^1^H-^15^N HSQC spectral changes of the dissociation intermediates at 310 K. The two spectra are displayed at equal contour levels. Assignments of cross-peaks attributed to dissociation intermediates and Ub^SH^ are colored red and black, respectively. (**b**) Real-time NMR profiles for intensity changes of the L67 cross-peaks of the dissociation intermediates (red) and Ub^SH^ (black) at 310 K. (**c**) Estimated activation energies (*E*_a_) for conversion of dissociation intermediates to Ub^SH^. Upper, middle, and lower dotted lines indicate average *E*_a_ (Ave) +1σ, Ave, and Ave −1σ, respectively. (**d**) Mapping of residues showing *E*_a_ < Ave −1σ (red) and *E*_a_ > Ave +1σ (blue) on the structure of ubiquitin (PDB database entry 1UBQ).

We found that the signal intensities of the minor cross-peaks decreased in a time-dependent manner (Fig. 4a,b), while those of the cross-peaks corresponding to Ub^SH^ increased exponentially in the same sample (Fig. 4a,b). These results indicate that the dissociation intermediates are thermodynamically unstable and are irreversibly converted to Ub^SH^ in a time-dependent manner.

**Figure 5:**
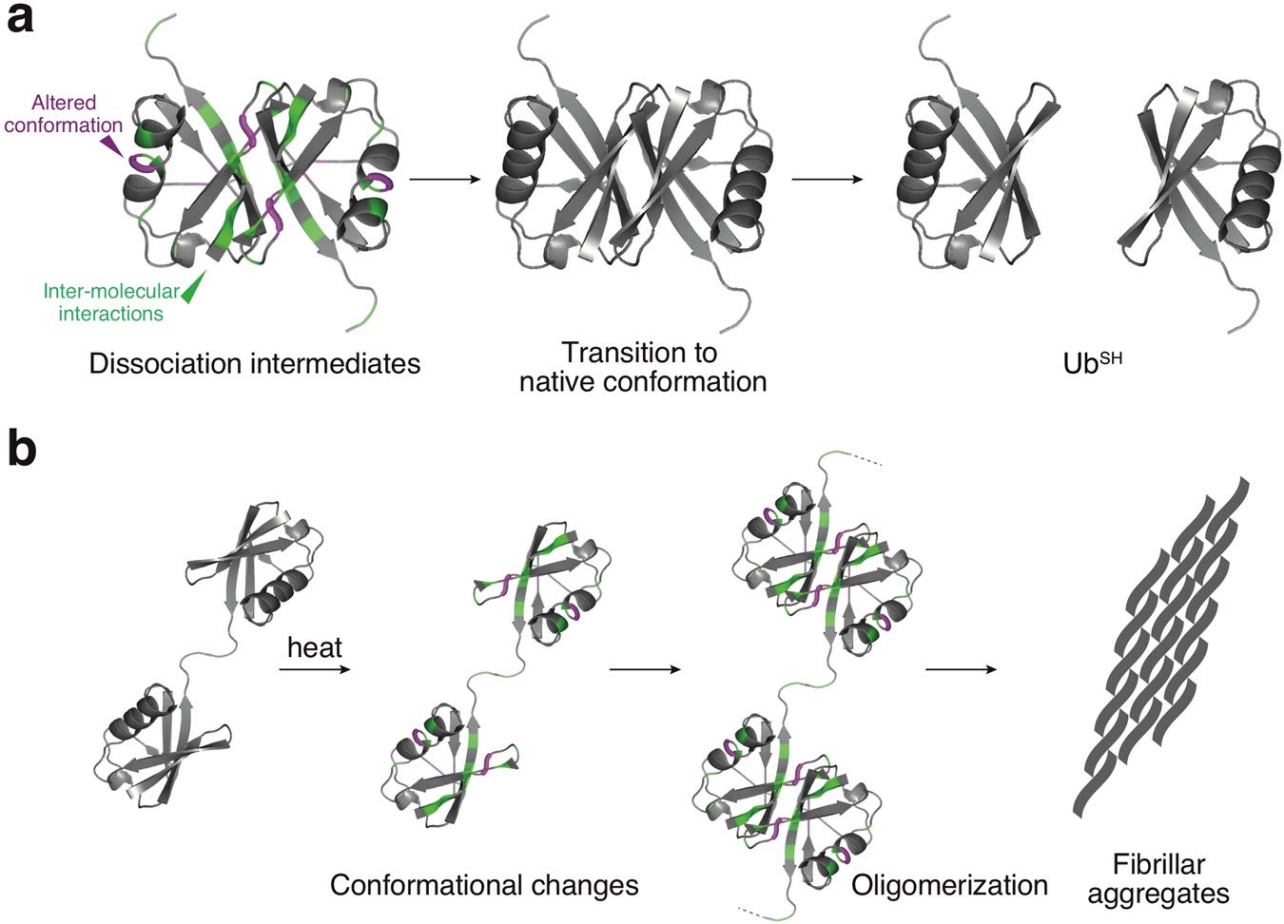
Proposed models of the dissociation and formation mechanism of polyubiquitin fibrils. (**a**) Model of the irreversible transition from the dissociation intermediates to Ub^SH^. Components in the dissociation intermediates associate with one another through the surfaces colored in green, as identified by chemical shift differences. In a time- and temperature-dependent transition to Ub^SH^, structural changes in the dissociation intermediates occur in the region colored in purple, where decreased secondary structure propensity was observed. This is followed by dissociation of the homodimer to Ub^SH^. (**b**) Model of diubiquitin fibril formation. Heat treatment induces partial structural changes in the ubiquitin moieties of diubiquitin and the structurally altered ubiquitin moieties associate with each other. The association leads to oligomerization and further fibril formation.

Therefore, we monitored the irreversible transition of the dissociation intermediates to Ub^SH^ at three different temperatures using real-time NMR (SI, Figs S8 and S9). Activation energies for the irreversible transitions were determined by fitting the increasing peak intensities of Ub^SH^ to first-order kinetics (see the Methods section: Estimation of activation energies). Activation energies were also estimated from the decreasing peak intensities of the dissociation intermediate (SI, Fig. S10); however, some of the values obtained were different from those estimated from the peak intensities of Ub^SH^. This may be due to an insufficient signal-to-noise ratio for data fitting (SI, Fig. S9); alternatively, the dissociation intermediate may convert to Ub^SH^ by more complicated kinetics. Notably, the average value of the activation energies estimated from the peak intensities of Ub^SH^ was found to be 118 kJ mol^−1^ (Fig. 4c), which is comparable to the activation energy of the dissociation of amyloid β protofibrils to monomers (80 kJ mol^−1^)^10^. In addition, we found that the activation energy was different for each residue (Fig. 4c). Because the activation energy is a kinetic parameter, the conversion is likely to initiate in regions corresponding to the lowest estimated activation energies. Therefore, the activation energies calculated by analysis of the peak intensities of Ub^SH^ indicate how the native structure of Ub^SH^ may be re-established from the dissociation intermediates. Relatively low activation energies were observed on three β-strands of ubiquitin (β3: R42 and I44; turn between β3 and β4: A46; β4: Q49; β5: T66) and on the second 3_10_-helix (D58 and N60) (Fig. 3b), whereas higher activation energies were estimated on the edges of β-strands (β1: K6; β2: I13; β5: L69), the α-helix (V26 and Q31), and the second 3_10_-helix (T55 and L56) (Fig. 4d). Together with the chemical shift analysis (Fig. 3a,b), these results argue that, in the conversion from dissociation intermediates to Ub^SH^, changes in the tertiary structure lead to subsequent dissociation of the homodimer (Fig. 5a).

## Discussion

Non-covalent associations between native ubiquitin molecules are reported to be very weak^11^. Likewise, here we observed no significant interaction between the ubiquitin moieties in Ub_2_^S-S^ (SI, Figs S2 and S3). However, inter-molecular interactions were present between ubiquitin molecules in the dissociation intermediates on the basis of size-exclusion chromatography (Fig. 1b and SI, Fig. S5) and the ^1^H-^15^N HSQC chemical shift analysis (Fig. 2). We observed structural differences between the dissociation intermediates and native ubiquitin (Fig. 3), and this structural alternation may play an important role in the non-covalent interactions associated with oligomerization and fibril formation. On the basis of our structural data, we propose that application of heat induces partial structural changes in the ubiquitin moieties, and these structurally altered ubiquitin moieties associate with each other non-covalently, ultimately leading to fibril formation (Fig. 5b). In the future, it will be interesting to examine the role of these observed structural changes and oligomerization in the formation of polyubiquitin fibrils.

In general, amyloid-forming proteins are thought to form soluble oligomeric intermediates such as oligomers, nuclei, and protofibrils before they form insoluble fibrils. Furthermore, the tertiary structures of oligomeric intermediates are thought to be distinguishable from those of the monomer and mature fibrils^12^; in addition, their cytotoxicity seems to be stronger than that of fibrils due to their high solubility and hydrophobicity^12^. Thus, to fully elucidate the structural and pathological properties of amyloid fibrils, it is important to acquire atomic-level information on their oligomeric states. As compared with intrinsically disordered amyloid-prone proteins such as amyloid β and α-synuclein, however, natively folded amyloid-forming proteins including β2-microglobulin and superoxide dismutase 1 tend to have more complicated oligomeric species along the amyloid fibril formation pathway^13^. In particular, tandem repeat proteins, such as the human muscle protein titin, have multiple intermediate species associated with amyloid fibril formation^14^. Polyubiquitin is a polymer of ubiquitin molecules, which, by analogy with titin, might infer the existence of multiple intermediate states along its pathway to fibril formation. If so, the dissociation intermediates observed in this study may reflect one of these intermediate states. It will be intriguing to determine the tertiary structure of this newly discovered fibril-derived molecule to reveal the details of its structural differences from native ubiquitin.

## Methods

### Protein preparation

Human ubiquitin G76C was expressed in *Escherichia coli* strain BL21 (*DE3*) in LB media (Nacalai Tesque) or M9 minimal media containing 99% ^15^N-labeled ammonium chloride (Cambridge Isotope Laboratories); for the triple-resonance NMR experiments, the M9 medium also contained 99% U-^13^C-labeled D-glucose (Cambridge Isotope Laboratories). The ubiquitin mutant was purified as described previously^5^. Protein purity was checked by SDS-PAGE.

### DTNB-assisted dimerization of ubiquitin G76C

Ubiquitin G76C mutant was reduced with 5 mM 2-mercaptoethanol (Nacalai Tesque) and then buffer-exchanged into 50 mM sodium phosphate buffer pH 7.5 using a PD-10 desalting column (GE Healthcare). The reduced ubiquitin mutant was mixed with a 20-fold molar excess of 5,5′-dithiobis-(2-nitrobenzoic acid) (DTNB, Tokyo Chemical Industry) and then incubated for ~2 hours at room temperature with linear shaking. The reaction solution was buffer exchanged into ligation buffer (20 mM Tris-HCl, 50 mM NaCl and 1 mM EDTA, pH 7.0) using a PD-10 desalting column. The reduced ubiquitin mutant was mixed with a 2-fold molar excess of the DTNB-activated ubiquitin mutant in which a disulfide bond had been established between the thiol groups of DTNB and the C-terminal cysteine of the ubiquitin G76C mutant; subsequently, this solution was incubated for 1 hour at room temperature with linear shaking. After residual DTNB was removed using a PD-10 desalting column, the resultant disulfide-conjugated diubiquitin (Ub_2_^S-S^) was separated from DTNB-activated and/or reduced ubiquitin mutants in 50 mM sodium phosphate and 150 mM NaCl, pH 7.0 by using a Hiload 16/60 Superdex 75 pg size exclusion column (GE Healthcare).

### Preparation of dissociation intermediates

To obtain Ub_2_^S-S^ fibrils, 1 mg ml^−1^ solution of Ub_2_^S-S^ was incubated at 368 K for 20 minutes in 50 mM sodium phosphate and 150 mM NaCl, pH 7.0. The solution was degassed, cooled to 277 K, before dithiothreitol (DTT, Nacalai Tesque) was added to a final concentration of 50 mM. The solution was then incubated at 277 K for more than 3 days, followed by centrifugation at 5,000 g for 10 minutes at 277 K and filtration using a 0.22 μm PVDF filter (Merck Millipore). The filtered sample was purified at 277 K in the presence of 5 mM DTT by using a Hiload 16/60 Superdex 75 pg size exclusion column (GE Healthcare). The first peak (elution volume of peak top: approximately 78 ml) in the size exclusion chromatography was collected as the dissociation intermediates.

### Fluorescence spectroscopy

Thioflavin T (ThT, Sigma Aldrich) and 8-anilino-1-naphthalenesulfonic acid (ANS, Sigma Aldrich) fluorescence was quantified on a FluoroMax4 (HORIBA) spectrometer at 277 K. ThT and ANS were excited at 440 nm and 335 nm, respectively; the emission spectra were acquired with a slit width of 5 nm in the range of 460–550 nm and 400–600 nm, respectively. Samples were diluted to a final concentration of 5 μM (defined as the concentration of one ubiquitin moiety) in 25 mM Tris-HCl, 150 mM NaCl, pH 8.0, and 25 μM ThT or 25 μM ANS. In the cases of Ub^SH^ and the dissociation intermediates, the buffer contained 5 mM DTT. The spectral contribution of the buffer was subtracted from the acquired spectra.

### Transmission electron microscopy

TEM images were obtained by using a JEM-1400Plus instrument (JEOL). The sample was loaded onto a collodion-coated grid and negatively stained with EM Stainer (Nisshin EM).

### NMR spectroscopy

All NMR spectra were acquired on an Avance 600 MHz or 700 MHz NMR spectrometer equipped with a 5 mm ^15^N/^13^C/^1^H z-gradient triple resonance cryoprobe (Bruker BioSpin). NMR samples were dissolved in 20 mM potassium phosphate, 20 mM KCl, 1 mM EDTA, 5 mM DTT, and 5% D_2_O (Cambridge Isotope Laboratories), pH 6.8. In the case of Ub_2_^S-S^, the buffer solution did not contain any reducing agent. For sequential backbone assignments, ^1^H-^15^N HSQC, HNCACB, and ^1^H-^15^N NOESY-HSQC spectra were acquired at 285 K. ^1^H chemical shifts were referenced to sodium 2,2-dimethyl-2-silapentane-5-sulfonate (DSS: Tokyo Chemical Industry) and both ^13^C and ^15^N chemical shifts were calibrated indirectly^15^. The concentration of ubiquitin moieties in the dissociation intermediates was 2 mM; the concentrations of Ub^SH^ and Ub_2_^S-S^ were 0.7 mM. Protein concentrations were determined by absorbance at 280 nm using NanoDrop 2000c (Thermo Fisher Scientific). To probe thermodynamic stability of the dissociation intermediates, ^1^H-^15^N SOFAST-HMQC^16^ spectra were acquired at 298, 308, and 310 K. Data processing was performed in NMRPipe^17^ and the data were analyzed in CcpNmr Analysis^18^.

### Estimation of activation energies

In the real-time NMR experiments, the signal intensities *I*(*t*, *T*) of each peak with measurement time *t* and temperature *T* (298, 304, and 310 K) were fitted to the equation *I*(*t*, *T*) = *I*_0_ exp[−*A*exp(−*E*_a_ *R*^−1^ *T*^−1^)*t*] + *C*, in which *I*_0_ is the initial signal intensities, *A* is the frequency factor, *E*_a_ is the activation energy, *R* is the gas constant, and *C* is a constant. Fitting was performed by using the program GLOVE^19^. Errors were calculated by the Monte Carlo method^19^. All fitting profiles are described in SI, Fig. S8 and S9.

### Mass spectrometry

The mass spectrum of Ub_2_^S-S^ was acquired in the reflector mode on an UltraflexIII matrix-assisted laser desorption ionization-time of flight/time of flight (MALDI-TOF/TOF) instrument (Bruker-Daltonics). One μl of sample was mixed with an equal volume of a saturated solution of 3,5-dimethoxy-4-hydroxycinnamic acid (Wako) in 50% acetonitrile (Nacalai Tesque) and 0.1% trifluoroacetic acid (Wako) on an MTP 384 target plate (Bruker-Daltonics). The theoretical monoisotopic mass was calculated by using the ChemCalc web application^20^.

## Electronic supplementary material

Supplementary Information
