# Supplementary material for: Isolation and characterization of a minimal building block of polyubiquitin fibrils
Source: Sci Rep. 2018 Feb 9;8:2711. doi: 10.1038/s41598-018-21144-z (PMC5807323; doi:10.1038/s41598-018-21144-z)
Supplement: Supplementary file 1 — Supplementary Information [file 41598_2018_21144_MOESM1_ESM.pdf]

Supporting Information

Isolation and characterization of a minimal building block of polyubiquitin fibrils

Daichi Morimoto, Erik Walinda, Mayo Shinke, Kenji Sugase, and Masahiro Shirakawa*

Supplementary Information

Supplementary Figures

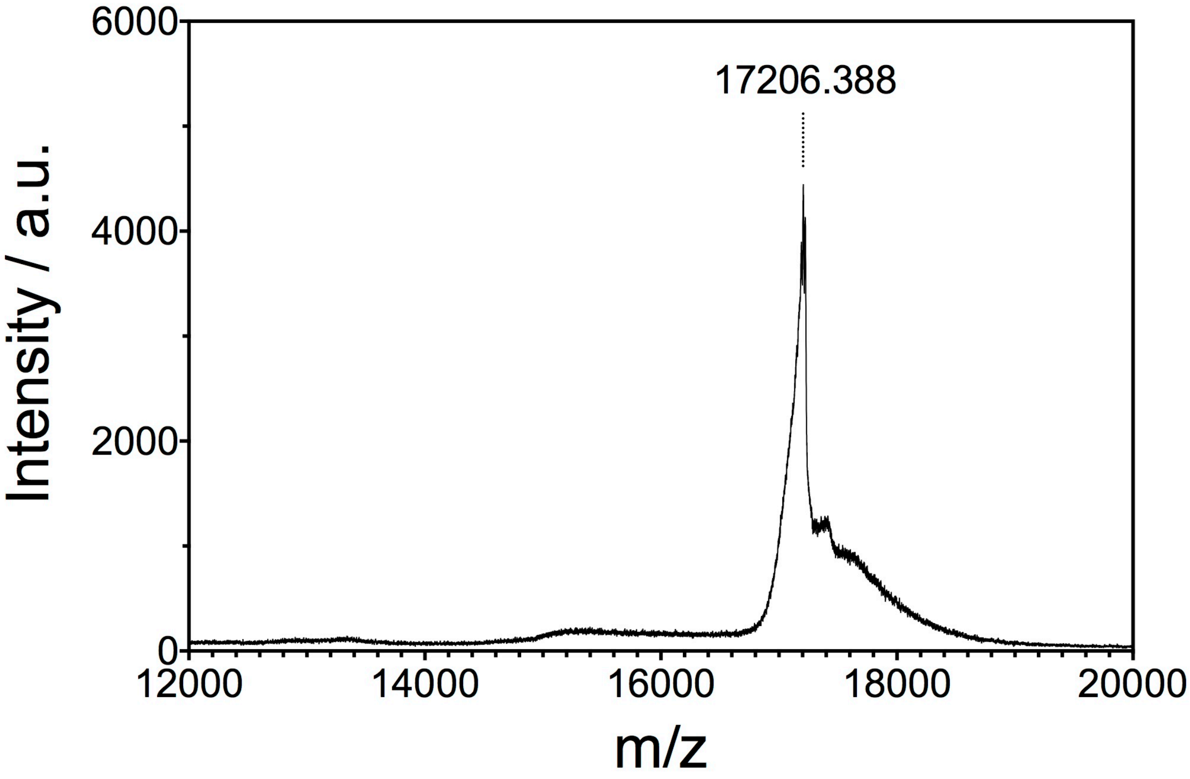

Figure S1. MALDI-TOF/TOF mass spectrum of disulfide-conjugated diubiquitin. The theoretical monoisotopic mass of disulfide-conjugated diubiquitin ($Ub_2^{S\text{-}S}$) is 17209.193. The difference between theoretical and experimental values was within the mass accuracy of the mass spectrometry instrument used in this study.

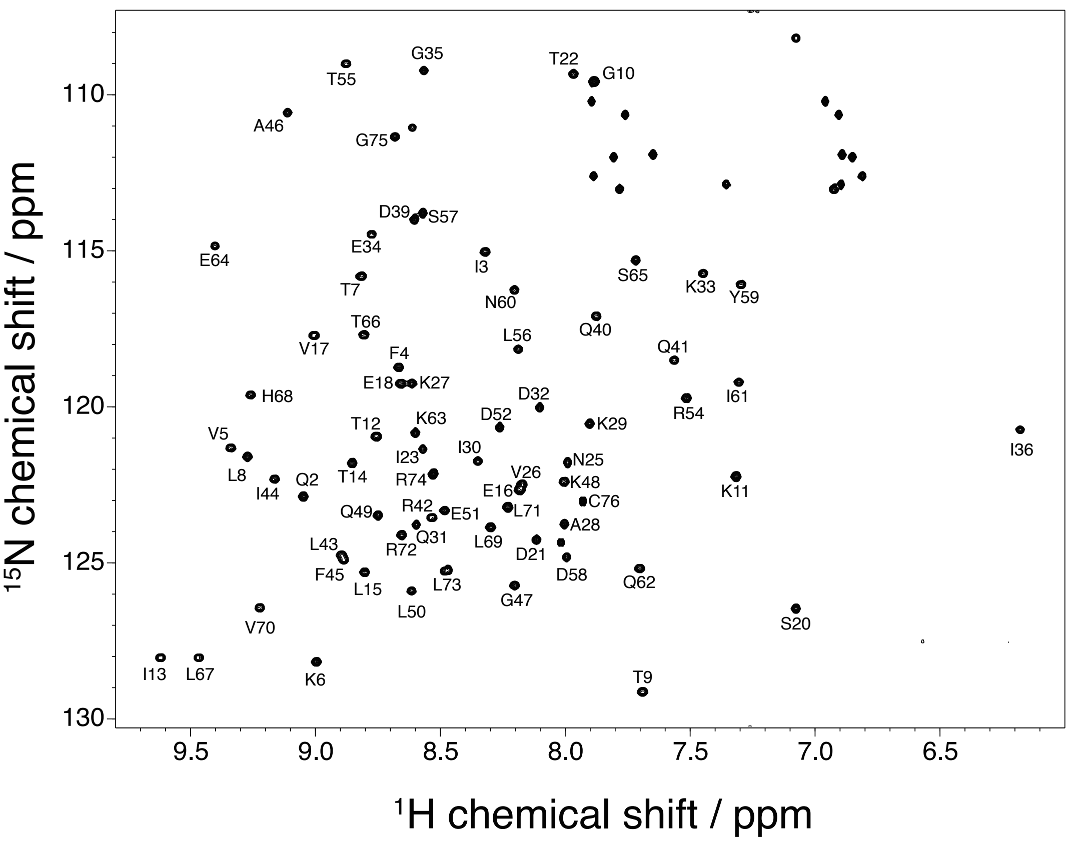

Figure S2. ^{1}H-^{15}N cross-peak assignment of ubiquitin (G76C). ^{1}H-^{15}N HSQC spectrum of the ubiquitin G76C mutant (Ub^{SH}) in the presence of 5 mM DTT at 285 K. The cross-peaks of T9, S20, A46, and G47 are aliased.

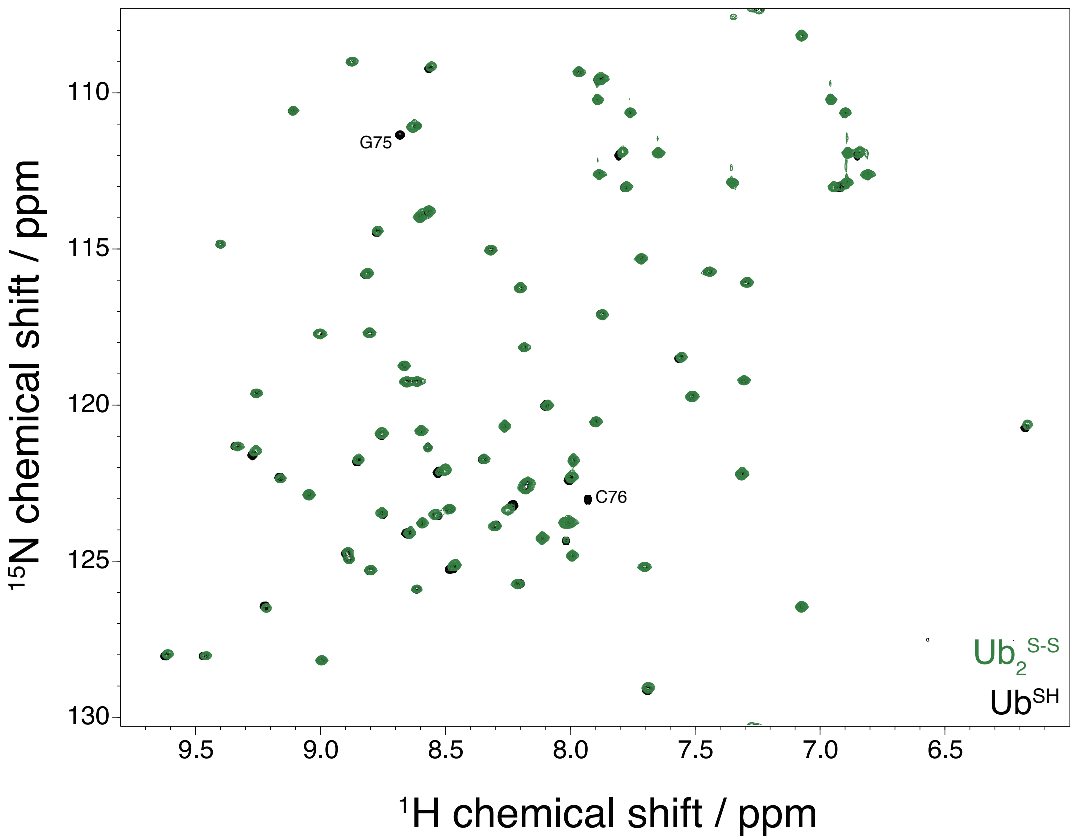

Figure S3. Subtle chemical shift differences between Ub^{SH} and $Ub_2^{S\text{-}S}$. ^{1}H-^{15}N HSQC spectra of Ub^{SH} (black) in the presence of 5 mM DTT and $Ub_2^{S\text{-}S}$ (green) in the absence of reducing agents at 285 K. Although the chemical shifts of the signals of the two C-terminal residues G75 and C76 displayed marked changes, no significant differences were observed for the remaining signals.

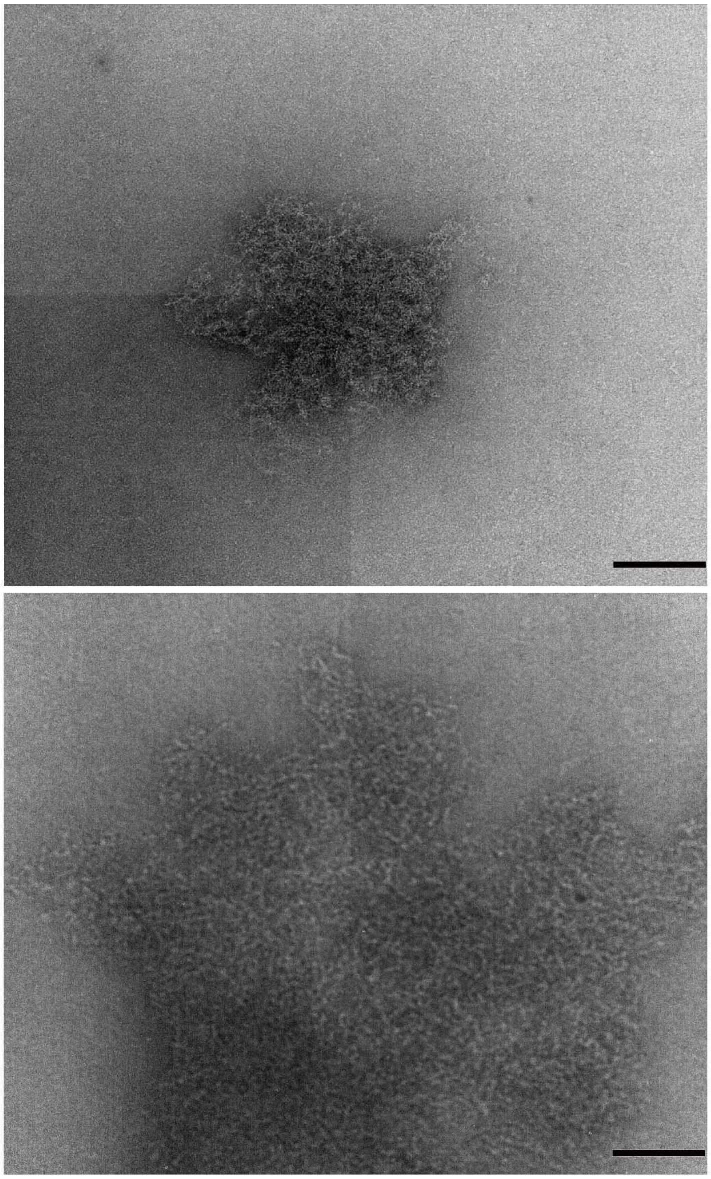

Figure S4. Fibrillar aggregates of disulfide-conjugated diubiquitin. Transmission electron microscopy images of heat-treated $Ub_2^{S\text{-}S}$. Bars, 200 nm.

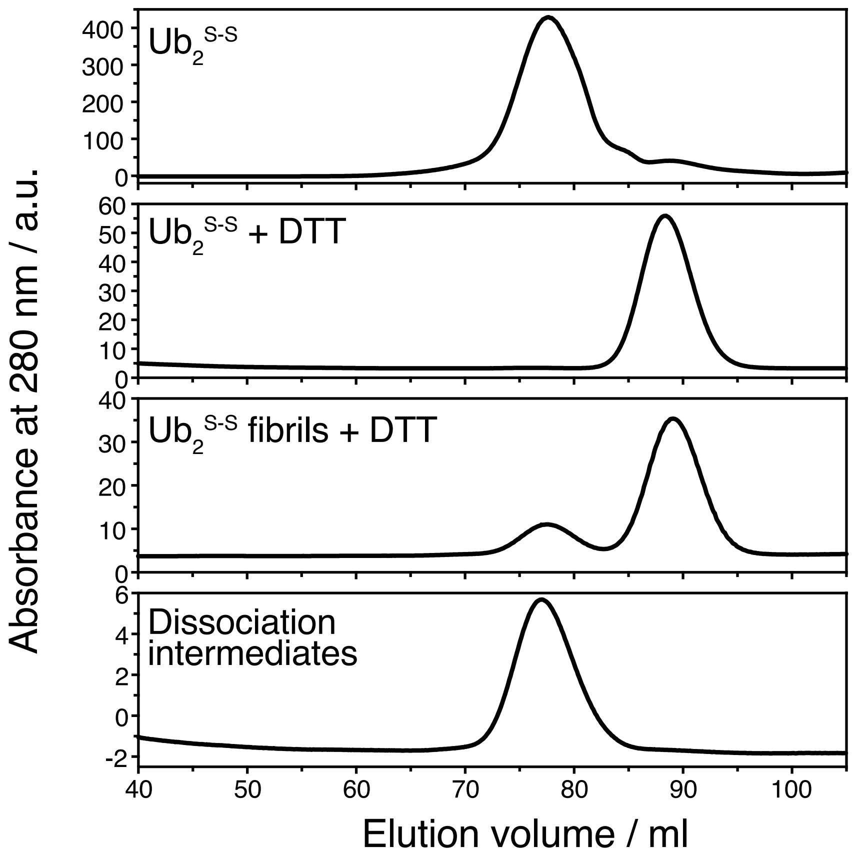

Figure S5. Size-exclusion chromatography analysis of diubiquitin and dissociation intermediates. Top, Ub$_2^{S\text{-}S}$ in the absence of reducing agents. Upper-middle, Ub$_2^{S\text{-}S}$ in the presence of 5 mM dithiothreitol (DTT). Ub$_2^{S\text{-}S}$ dissociated to Ub^{SH}. Lower-middle, the solution obtained by treatment of Ub$_2^{S\text{-}S}$ fibrils with 5 mM DTT. Bottom, dissociation intermediates in the presence of 5 mM DTT.

	Ub^SH			
Residue	HN /ppm	N /ppm	C_α /ppm	C_β /ppm
1				
2	9.04	122.79	54.90	30.68
3	8.32	114.96	59.65	42.20
4	8.66	118.67	55.07	41.28
5	9.33	121.25	60.34	34.42
6	8.99	128.11	54.72	34.34
7	8.81	115.73	60.58	70.61
8	9.27	121.51	57.58	42.02
9	7.69	106.05	61.42	69.13
10	7.88	109.50	45.42	
11	7.31	122.16	56.37	33.48
12	8.75	120.88	62.53	69.71
13	9.62	127.98	59.93	40.76
14	8.85	121.74	62.10	69.64
15	8.80	125.23	52.81	46.92
16	8.18	122.61	54.83	29.75
17	9.00	117.64	58.38	36.50
18	8.65	119.18	52.80	30.93
19			65.32	31.87
20	7.07	103.39	57.48	63.44
21	8.11	124.19	55.91	40.85
22	7.96	109.25	59.69	71.23
23	8.57	121.28	62.40	34.57
24			60.84	28.82
25	7.99	121.71	56.12	38.50
26	8.17	122.47	67.71	30.76
27	8.61	119.18	59.29	33.73
28	8.00	123.68	55.38	17.71
29	7.90	120.46	59.78	33.39
30	8.35	121.68	66.16	36.86
31	8.59	123.70	59.99	27.79
32	8.10	119.95	57.54	40.96
33	7.44	115.67	58.11	34.08
34	8.77	114.38	55.29	33.52
35	8.56	109.14	46.01	
36	6.17	120.65	57.92	40.61
37				
38			66.14	32.85
39	8.60	113.91	55.88	39.79
40	7.87	117.04	55.65	30.09
41	7.56	118.41	56.65	31.47
42	8.53	123.49	55.15	31.67
43	8.89	124.70	53.15	45.83
44	9.16	122.26	58.91	41.12
45	8.88	124.83	56.52	43.77
46	9.11	133.50	52.55	16.58
47	8.20	102.66	45.35	
48	7.99	122.29	54.67	34.46
49	8.75	123.41	56.10	29.19
50	8.61	125.83	54.19	41.39
51	8.48	123.25	55.93	31.93
52	8.26	120.60	56.14	40.68
53			45.19	
54	7.51	119.64	54.38	32.78
55	8.87	108.93	59.66	72.39
56	8.18	118.09	58.61	40.28
57	8.56	113.72	61.20	62.43
58	7.99	124.76	57.49	40.34
59	7.29	115.99	58.16	40.10
60	8.20	116.17	54.21	37.36
61	7.30	119.13	62.55	36.88
62	7.70	125.12	53.56	31.68
63	8.59	120.75	57.92	32.56
64	9.40	114.78	58.10	25.98
65	7.71	115.23	61.05	64.97
66	8.80	117.62	62.65	70.36
67	9.46	127.97	53.70	44.45
68	9.25	119.57	56.39	32.58
69	8.30	123.77	53.71	44.09
70	9.22	126.40	60.53	34.95
71	8.23	123.19	54.17	42.69
72	8.65	124.04	55.72	31.41
73	8.47	125.14	54.86	42.44
74	8.52	122.06	56.60	30.80
75	8.68	111.27	45.46	
76	7.93	122.95	59.50	29.29

	Fibril-derived molecules			
Residue	HN /ppm	N /ppm	C_α /ppm	C_β /ppm
1				
2	9.02	123.79	54.98	30.51
3	8.45	116.12	59.80	42.30
4	8.78	118.89	55.02	41.10
5	9.23	120.25	59.87	35.29
6	8.83	128.23	54.93	33.07
7	8.92	116.30	61.20	69.84
8	9.22	121.80	57.56	42.04
9	7.59	105.09	61.53	69.01
10	7.82	109.33	45.45	
11	7.25	121.49	56.03	33.62
12	8.73	120.01	62.86	69.25
13	9.45	125.90	59.48	40.79
14	8.82	121.06	62.17	69.74
15	8.86	125.45	52.84	46.65
16				
17				
18				
19			65.30	31.80
20	7.03	103.31	57.40	63.49
21	8.06	124.30	55.84	40.95
22	8.13	109.66	59.74	71.00
23				
24				
25	7.95	121.33	56.04	38.68
26	8.13	122.37	67.72	30.95
27	8.54	118.24	60.54	32.46
28	7.80	121.85	55.04	17.98
29	7.96	119.12	59.18	33.44
30	8.27	121.52	66.17	36.89
31				
32	7.94	119.78	57.65	41.15
33	7.36	115.46	57.97	34.04
34			55.26	33.48
35	8.61	109.06	46.01	
36	6.15	120.40	57.71	40.59
37				
38			66.32	32.86
39	8.64	113.90	55.89	39.83
40	7.84	117.04	55.64	30.10
41	7.50	118.23	56.59	31.49
42				
43	8.87	124.29	53.25	45.80
44	9.21	122.47	59.31	40.80
45			56.29	43.72
46	9.21	133.84	52.43	16.45
47	8.13	102.78	45.36	
48	8.03	122.60	54.73	34.44
49	8.81	124.28	55.63	29.34
50	8.61	126.18	54.22	41.45
51	8.39	123.43	55.90	31.89
52				
53			45.32	
54	7.46	119.69	54.53	32.65
55	8.83	108.43	59.65	72.34
56	8.04	118.13	58.72	40.48
57	8.66	113.77	61.12	62.32
58	7.86	124.43	57.59	40.46
59	7.34	116.15	58.19	40.14
60	8.17	116.53	54.07	37.52
61	7.23	118.87	62.36	36.94
62	7.83	125.05	53.59	31.47
63	8.65	120.79	57.86	32.67
64	9.35	114.32	58.00	25.89
65	7.65	114.69	60.68	65.03
66	8.84	117.84	62.65	70.24
67	9.32	126.04	53.02	44.67
68	9.05	118.70	55.89	33.20
69	8.23	123.96	54.24	44.16
70	9.23	125.43	60.29	34.86
71	8.15	123.21	54.20	42.69
72	8.58	124.64	55.75	31.47
73	8.44	125.76	54.81	42.41
74	8.50	121.97	56.65	30.78
75	8.11	109.20	45.26	
76				

Figure S6. Chemical shift assignments for Ub^{SH} and the dissociation intermediates. ^{1}H chemical shifts were referenced to sodium 2,2-dimethyl-2-silapentane-5-sulfonate (DSS) and both ^{13}C and ^{15}N chemical shifts were calibrated indirectly.

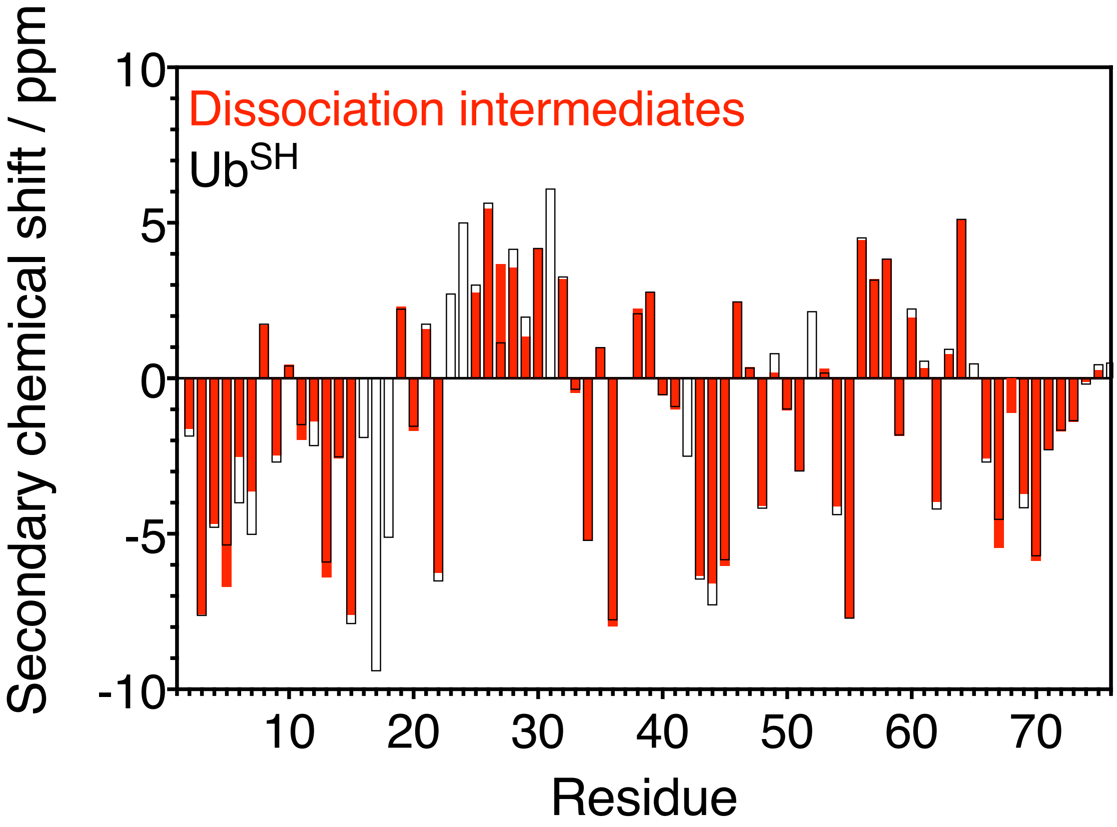

Figure S7. Secondary chemical shifts of C_α and C_β for the dissociation intermediates and Ub^{SH}. Secondary chemical shifts are obtained as $\Delta\delta_{C\alpha} - \Delta\delta_{C\beta}$, where $\Delta\delta_{C\alpha}$ and $\Delta\delta_{C\beta}$ are the differences in C_α and C_β chemical shifts between the observed and random-coil chemical shift values. Red bars, dissociation intermediates; white bars, Ub^{SH}.

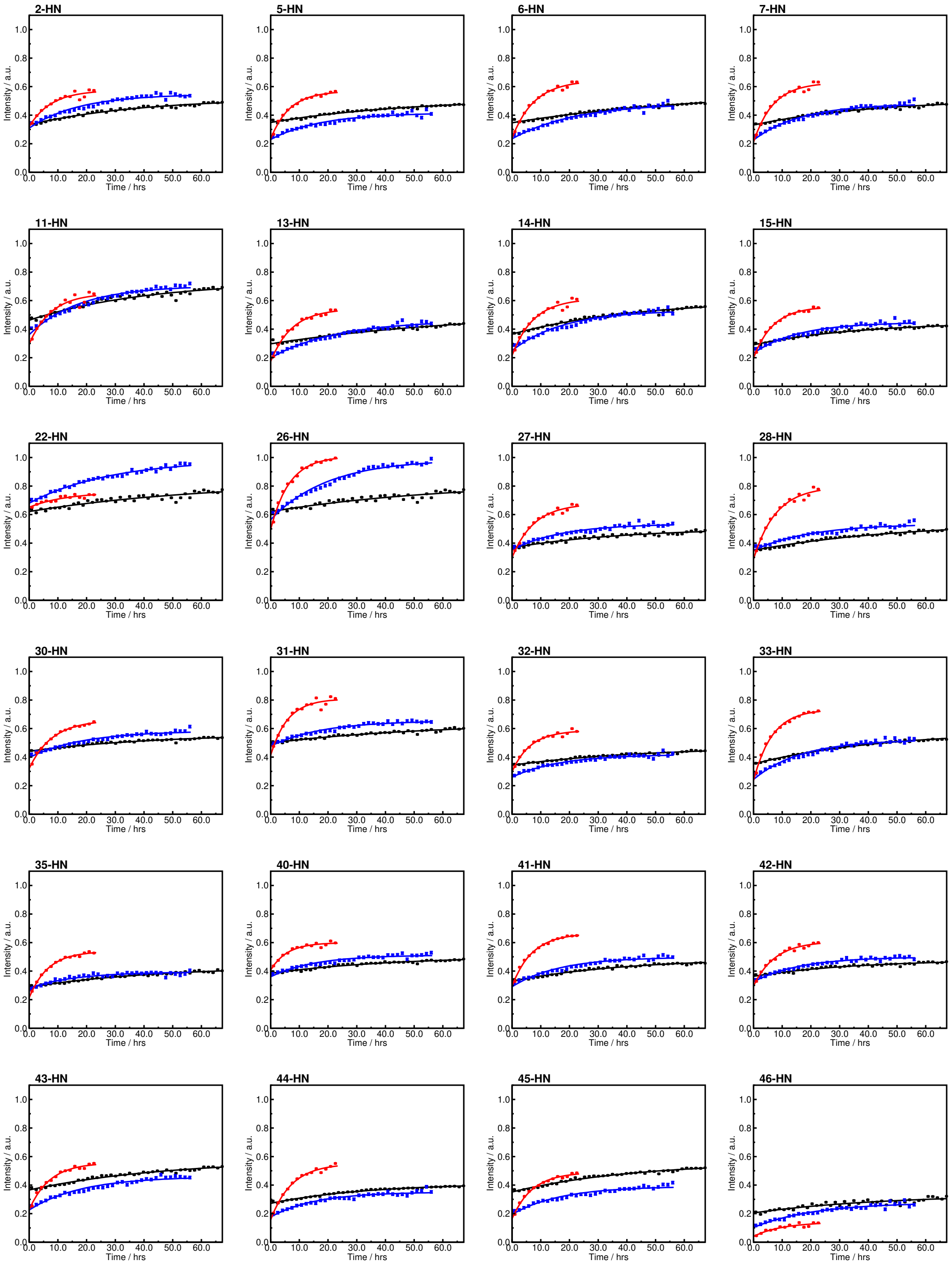
2-HN
5-HN
6-HN
7-HN
11-HN
13-HN
14-HN
15-HN
22-HN
26-HN
27-HN
28-HN
30-HN
31-HN
32-HN
33-HN
35-HN
40-HN
41-HN
42-HN
43-HN
44-HN
45-HN
46-HN
Intensity / a.u.
Time / hrs

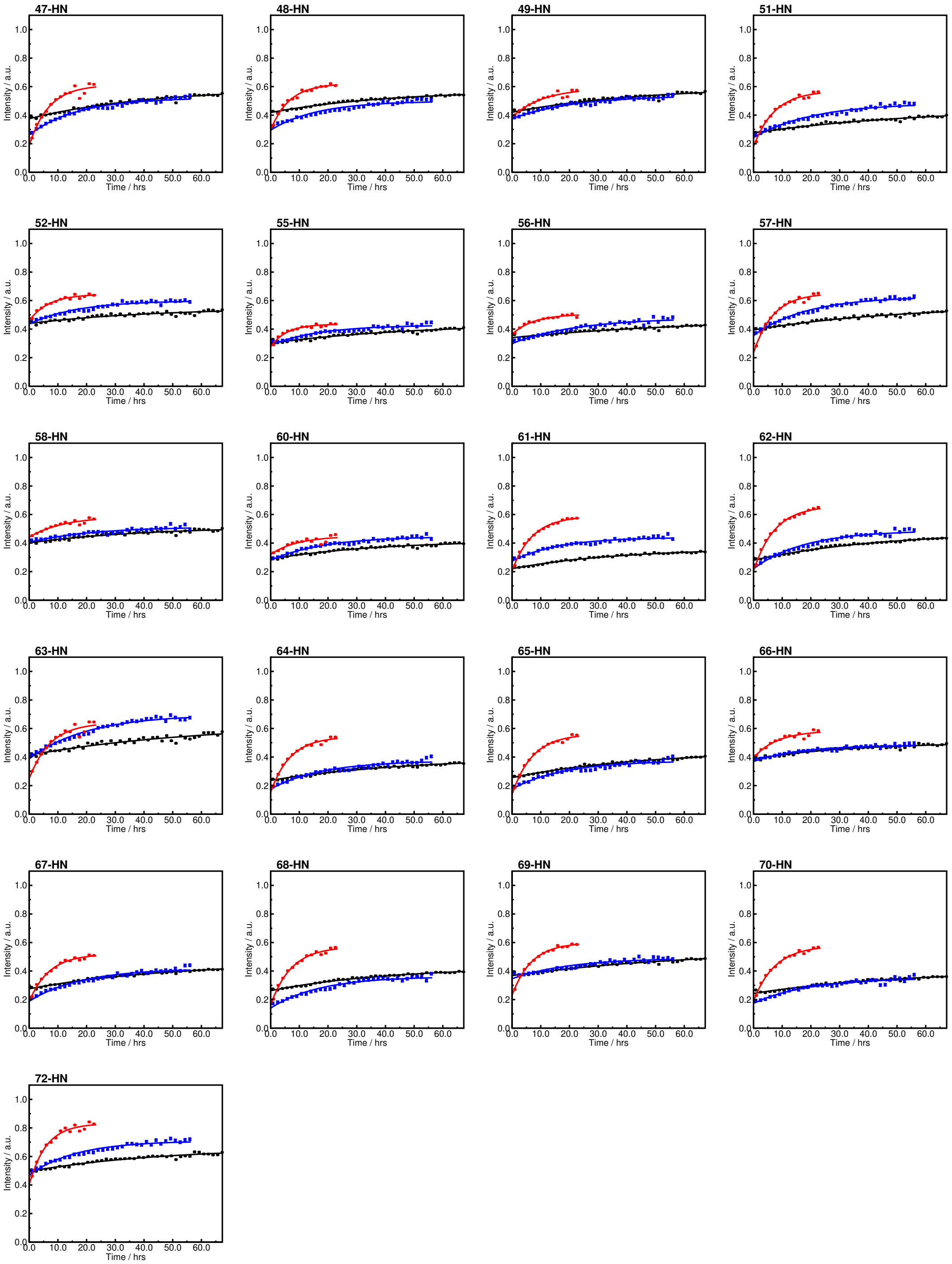

Figure S8. Time- and temperature-dependent peak intensity increases in the Ub^{SH} cross-peak intensities due to the conversion of dissociation intermediates to Ub^{SH}. Real-time NMR profiles of ^{1}H-^{15}N cross-peaks attributed to Ub^{SH} at 298 K (black), 308 K (blue), and 310 K (red). Error bars, standard deviation as obtained by Monte-Carlo simulation (100 iterations).

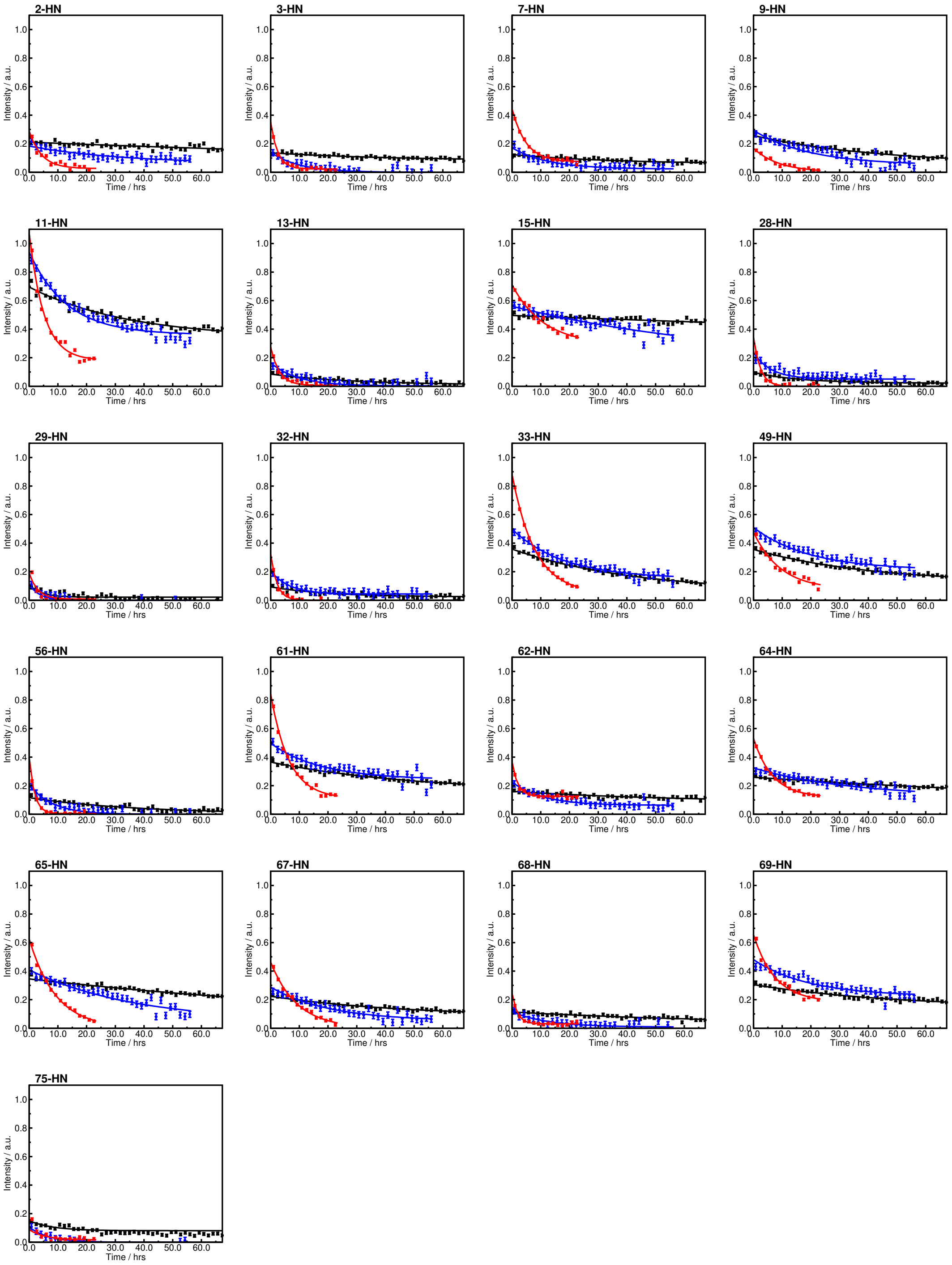

Figure S9. Time- and temperature-dependent peak intensity decreases in the dissociation intermediate cross-peak intensities due to the conversion of dissociation intermediates to Ub^{SH}. Real-time NMR profiles of ^{1}H-^{15}N cross-peaks attributed to dissociation intermediates at 298 K (black), 308 K (blue), and 310 K (red). Error bars, standard deviation as obtained by Monte-Carlo simulation (100 iterations).

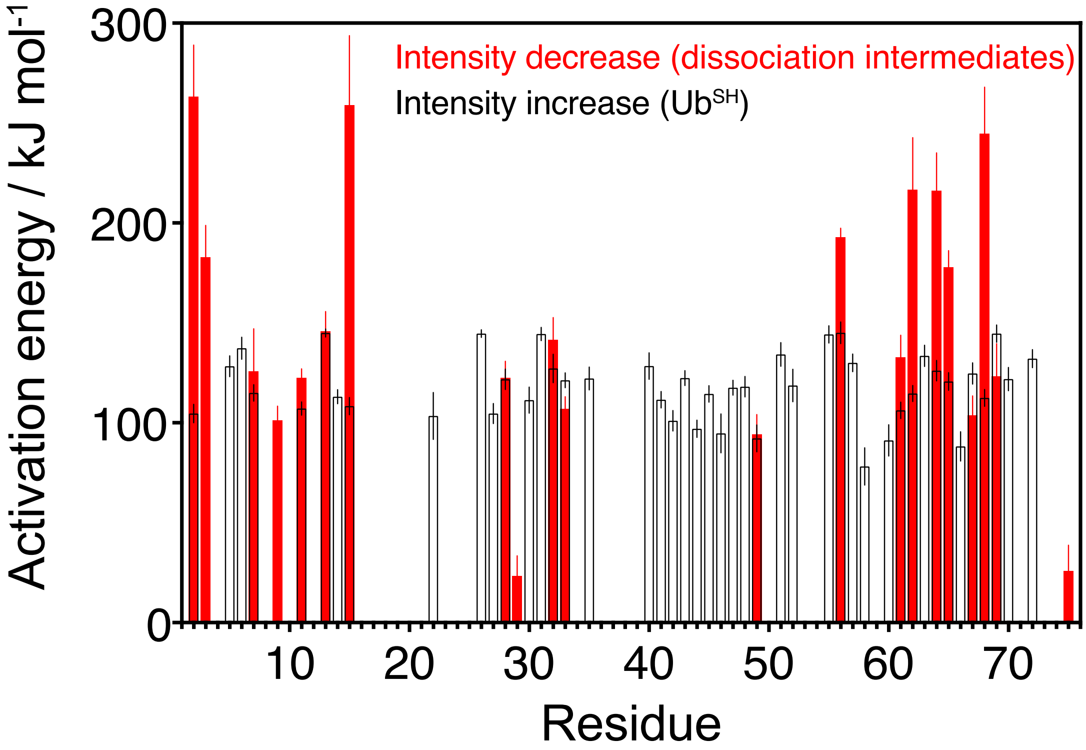

Figure S10. Activation energies for conversion of the dissociation intermediates to Ub^{SH}. The activation energies are calculated by fitting of the peak intensities of dissociation intermediates (red) and Ub^{SH} (white) to first-order kinetics (see the Method section: Estimation of activation energies). Red bars, dissociation intermediates; white bars, Ub^{SH}. Error bars, standard deviation as obtained by Monte-Carlo simulation (100 iterations).
